# Intelligent Biosensors for Diabetic Wound Monitoring

**DOI:** 10.3390/bios16060307

**Published:** 2026-05-26

**Authors:** Shuqin Li, Xiu-Hong Wang

**Affiliations:** 1 Laboratory for Biomedical Photonics, Institute of Laser Engineering, School of Physics and Optoelectronic Engineering, Beijing University of Technology, Beijing 100124, China; 2 Key Laboratory of Trans-Scale Laser Manufacturing Technology, Ministry of Education, Beijing 100124, China; 3 Beijing Engineering Research Center of Laser Technology, Beijing 100124, China

**Keywords:** diabetic wounds, smart dressings, in situ monitoring, machine learning

## Abstract

Diabetic chronic wounds, characterized by persistent inflammation and a complex microenvironment, pose a major challenge to global healthcare. Traditional dressings act merely as passive physical barriers, lacking the ability to sense biochemical fluctuations or respond to dynamic pathological changes. Therefore, developing smart platforms for in situ, continuous, and non-invasive monitoring is crucial for early warning and precision intervention. This review systematically explores recent advances in high-fidelity wound monitoring, focusing on the deep integration of “front-end interface engineering” and “back-end data analysis”. We first analyze the specific physicochemical and biochemical abnormalities of the diabetic wound microenvironment. Next, we discuss how advanced material designs, such as active fluid management, anti-biofouling zwitterionic networks, and nanozyme-based reactive oxygen species (ROS) scavenging, ensure the long-term stability of sensing interfaces against complex microenvironmental interference. Building on this hardware foundation, we summarize in situ sensing strategies and multiparameter decoupling techniques tailored for key biomarkers, including pH, temperature, glucose, ROS, and MMP-9. Furthermore, we highlight cutting-edge developments in signal digitization, emphasizing the pivotal role of portable devices and machine learning algorithms in extracting high-dimensional features and translating complex multimodal signals into objective clinical metrics. By outlining this comprehensive technological closed-loop, this review aims to provide a systematic theoretical framework for the development and clinical translation of next-generation smart wound monitoring platforms.

## 1. Introduction

Diabetes currently affects 463 million people worldwide, with projections rising to 700 million by 2045. Diabetic foot ulcers develop in 7.2–15% of these patients. Among them, 50–60% progress to secondary infections, and approximately one-fifth of these infections are moderate to severe, often leading to lower limb amputation [1]. Diabetic chronic wounds represent one of the most severe and prevalent complications of the escalating global diabetes epidemic. Due to their refractory nature and the high risk of adverse outcomes, they pose a formidable challenge to public health [2,3,4,5,6]. Generally, wound healing is a highly orchestrated biological process encompassing hemostasis, inflammation, proliferation, and tissue remodeling. However, in diabetic patients, this sophisticated healing cascade is disrupted by an extremely hostile microenvironment, leading to the formation of recalcitrant diabetic chronic wounds [7,8]. Unlike the transient inflammation observed in acute wounds, diabetic wounds remain stalled in a persistent inflammatory state driven by a complex interplay of pathological factors; these include the accumulation of hyperglycemia-induced advanced glycation end products (AGEs), excessive oxidative stress (ROS), and a proteolytic imbalance characterized by the overexpression of proteases [7,9,10,11,12,13,14]. These biochemical disturbances further trigger physical alterations, such as alteration in pH from acidic to alkaline, creating a microenvironment susceptible to bacterial infection and biofilm formation [15,16]. Consequently, this multifactorial healing arrest not only hinders the transition of the wound into the proliferative phase but also imposes a heavy burden of amputations and healthcare costs globally [5,6,9,17,18].

Facing this complex and dynamically evolving microenvironment, traditional wound dressings (such as gauze and films) are limited to acting as passive physical barriers and absorbing exudates. They lack the inherent capacity to sense biochemical fluctuations or respond in real-time to pathological changes [1,17]. Clinicians typically must assess wounds through frequent dressing removal, which not only heightens patient discomfort but also predisposes the site to secondary infections and risks damaging fragile nascent granulation tissue. Consequently, the development of intelligent platforms capable of in situ, continuous, and non-invasive monitoring of the wound microenvironment is of paramount clinical significance for achieving early warning and precision intervention.

In recent years, advancements in flexible wearable electronics and biomaterials have introduced novel interventional strategies for the aforementioned challenges. Integrating miniaturized optical and electrochemical sensors into wound dressings allows for the continuous monitoring of critical parameters, including physicochemical factors (like pH and temperature) and biochemical markers (such as glucose, reactive oxygen species (ROS), and MMP-9). Despite this progress, the clinical translation of such technology still encounters primary bottlenecks: first, the instability of the sensing interface and subsequent signal drift caused by the complex microenvironment (e.g., high exudate levels, mechanical stress, and biofouling); second, the difficulty in effectively extracting clinical diagnostic indicators from high-dimensional, multimodal signals. Consequently, research in this field has progressively expanded to include the development of anti-fouling stable interfaces, multi-signal decoupling, and intelligent data analysis fused with machine learning.

In this context, intelligence in wound monitoring encompasses interconnected dimensions: interface intelligence, which refers to the ability of materials to actively adapt to the wound environment through anti-biofouling properties, self-healing capabilities, and stimuli-responsive behaviors; sensing intelligence, which involves the capacity for multimodal signal decoupling, wireless transmission, and on-site data processing without external instrumentation; and diagnostic intelligence, which leverages machine learning algorithms to transform complex sensor outputs into objective, clinically actionable metrics. Together, these three dimensions constitute the core framework for transitioning from passive monitoring to truly intelligent wound management.

At present, the majority of literature regarding smart dressings focuses on the validation of specific sensing mechanisms or the synthesis of novel materials, whereas the integration of “front-end interface engineering” and “back-end data analysis” remains insufficiently explored. To bridge this gap, this review, guided by the pathological microenvironment of diabetic wounds (Section 2), systematically delineates the core trajectory for achieving high-fidelity in situ monitoring. Specifically, it examines how material-level design can withstand extreme microenvironmental interference to maintain the long-term stability of the sensing interface (Section 3). Building upon this hardware foundation, we detail in situ sensing and multi-parameter integration strategies developed for key pathological biomarkers (Section 4). Furthermore, this work explores cutting-edge progress in leveraging portable devices and machine learning algorithms to transform complex multimodal signals into objective clinical diagnostic indicators (Section 5). By outlining this comprehensive technical closed-loop, we aim to provide a systematic theoretical framework for the development and clinical translation of next-generation smart platforms for wound diagnostics.

## 2. The Healing Process and Microenvironment of Diabetic Wounds

Developing intelligent diagnostic and therapeutic platforms requires a precise understanding of the localized wound pathology. In diabetic chronic wounds, the physiological repair process is fundamentally dysregulated and stagnant. Analyzing these aberrant characteristics and identifying key microenvironmental markers across healing stages provides the theoretical foundation for establishing the sensing targets and intervention strategies of smart dressings. In this review, diabetic ulcers refer specifically to diabetic foot ulcers (DFUs), which face the unique challenge of weight-bearing pressure—an additional mechanical stress that impairs healing and increases the risk of chronicity.

### 2.1. Physiology of Wound Healing and Pathological Changes in Diabetes

**Figure 1:**
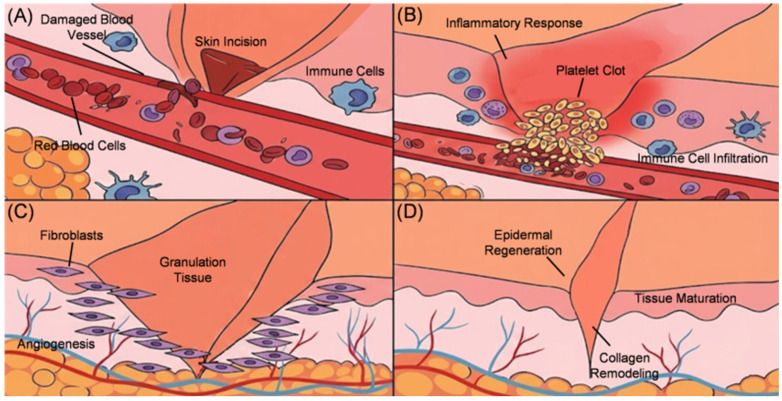
The four stages of wound healing: (**A**) hemostasis, (**B**) inflammation, (**C**) proliferation, and (**D**) remodeling. (Solid lines denote structural labels. Gemini 3.0 pro was used to generate certain elements of this figure).

Physiological skin wound healing is a highly orchestrated biological process comprising four sequential stages: hemostasis, inflammation [19], proliferation [20], and tissue remodeling [21] (Figure 1). Upon skin injury, platelet aggregation initiates the coagulation cascade to achieve hemostasis. This is followed by a rapid transition into the inflammatory phase, where neutrophils and monocytes are recruited to the injury site and differentiate into macrophages. These cells release reactive oxygen species (ROS) and proteases to eliminate pathogens while secreting cytokines to trigger tissue regeneration. Subsequently, the wound enters the proliferative phase, during which fibroblasts and keratinocytes generate granulation tissue, deposit collagen, and facilitate neovascularization. Finally, as an equilibrium is attained between collagen synthesis and degradation, the extracellular matrix (ECM) matures, and the wound undergoes functional repair [22,23,24,25,26,27].

However, under pathological conditions such as diabetes, this highly orchestrated healing cascade is profoundly disrupted. Diabetes mellitus stands as one of the most prominent systemic diseases contributing to impaired wound healing. The persistent hyperglycemic state not only triggers neuropathy and vasculopathy but also imposes significant impediments across all critical stages of the wound-healing process:

In the hemostasis phase, diabetes disrupts the homeostatic balance between blood coagulation and fibrinolysis. The wound microenvironment shifts into a prothrombotic and hypercoagulable state. Hyperglycemia induces platelet hyperreactivity, leading to excessive aggregation at the site of vascular injury, while abnormally active coagulation factors result in the formation of an overly dense fibrin network. Simultaneously, the body’s capacity for fibrinolysis is severely impaired. This milieu leads to the formation of persistent, dissolution-resistant microthrombi within the microvasculature. These thrombi obstruct local blood circulation, hindering the delivery of oxygen and nutrients. Ultimately, this prolongs the hemostasis stage, preventing the healing process from progressing successfully [28].

Moving to the inflammatory phase, hyperglycemia-induced oxidative stress severely disrupts the normal physiological functions of macrophages. This interference prevents the essential polarization of macrophages from a pro-inflammatory M1 phenotype to a pro-healing M2 phenotype. Such an aberrant response leads to the localized accumulation of abundant pro-inflammatory cytokines and destructive proteases, while simultaneously hindering the secretion of pro-healing growth factors. Consequently, the wound becomes trapped in a state of persistent and excessive inflammation [29]. This prolonged inflammatory milieu severely compromises the progression of subsequent healing phases, obstructing the transition into the proliferative stage and ultimately causing the healing process to stagnate into a chronic, non-healing wound.

In the proliferative phase, hyperglycemia severely interferes with the normal formation of granulation tissue. The proliferation and migration capabilities of fibroblasts are significantly diminished, and these cells exhibit a blunted responsiveness to growth factors. Simultaneously, fibroblasts produce excessive proteases that degrade the connective tissue. The growth and tube formation of vascular endothelial cells are also markedly hindered; the wound lacks sufficient angiogenic factors, leading to inadequate local blood supply. Additionally, epithelial cells lose their ability to migrate toward the center of the wound. Collectively, these obstacles make wound closure difficult and severely reduce overall healing efficiency [30].

During the remodeling phase, diabetic wound healing continues to face numerous obstacles. First, there is an imbalance in matrix degradation. Due to the abnormal expression of degradative enzymes and fibrinolysis inhibitors, excessive amounts of collagen and fibronectin accumulate locally, which sequesters and inactivates essential repair growth factors. Second, persistent hyperglycemia triggers the accumulation of advanced glycation end products (AGEs), leading to the pathological cross-linking of collagen fibers. This hinders the rearrangement and maturation of collagen, resulting in scar tissue that lacks sufficient flexibility and tensile strength. Meanwhile, the thickening of the vascular basement membrane in the microcirculation interferes with nutrient and oxygen exchange, delaying functional tissue reconstruction [31]. While ideal healing should promote structural remodeling and functional recovery, the low-quality matrix formed under diabetic conditions leaves the closed wound fragile and prone to post-healing re-ulceration.

This systemic and full-cycle healing arrest is rooted in the extreme deterioration of the localized wound microenvironment. Therefore, an in-depth dissection of the specific pathological features of the diabetic wound microenvironment is essential to revealing the underlying mechanisms of its recalcitrant nature.

### 2.2. Physicochemical and Biochemical Characteristics of the Diabetic Wound Microenvironment

The development of chronic diabetic wounds is the cumulative result of multifaceted biochemical metabolic disturbances and the imbalance of physicochemical factors. In contrast to physiological wounds, the diabetic wound microenvironment exhibits profoundly exacerbated pathological features, which subsequently define precise targets for real-time monitoring and therapeutic intervention.

#### 2.2.1. Biochemical and Metabolic Derangements

Biochemical and metabolic derangements are fundamental factors contributing to the impaired healing of diabetic wounds. Hyperglycemia, the most prominent hallmark of diabetic wounds, readily leads to the accumulation of advanced glycation end products (AGEs). These AGEs hinder wound healing by increasing apoptosis, reducing fibroblast proliferation, and diminishing the bioactivity of fibroblast growth factors (FGF). Simultaneously, AGEs promote macrophage polarization toward the classically activated phenotype (M1 macrophages), resulting in a massive release of pro-inflammatory cytokines [25,26,27,32]. This hyperglycemic milieu further induces elevated levels of oxidative stress. Elevated reactive oxygen species (ROS) weaken antioxidant defenses, inhibit the secretion of cytokines and growth factors, and obstruct the formation of fibroblasts, collagen fibers, and neovascularization. Furthermore, abnormal lipid metabolism promotes the release of inflammatory mediators, thereby inducing the infiltration of macrophages and other immune cells [33,34,35]. The extensive recruitment of inflammatory cells significantly increases oxygen consumption at the wound site, leading to severe localized hypoxia and ischemia, which subsequently impairs collagen synthesis, angiogenesis, and re-epithelialization [36,37,38]. Additionally, this hostile environment disrupts proteolytic balance, resulting in the overexpression of matrix metalloproteinases (MMPs) that degrade essential tissue-repair proteins. This process exacerbates tissue necrosis, accompanied by significant protein exudation, ultimately delaying wound closure or resulting in a chronic non-healing state [39].

#### 2.2.2. Physicochemical Microenvironmental Characteristics

**Figure 2:**
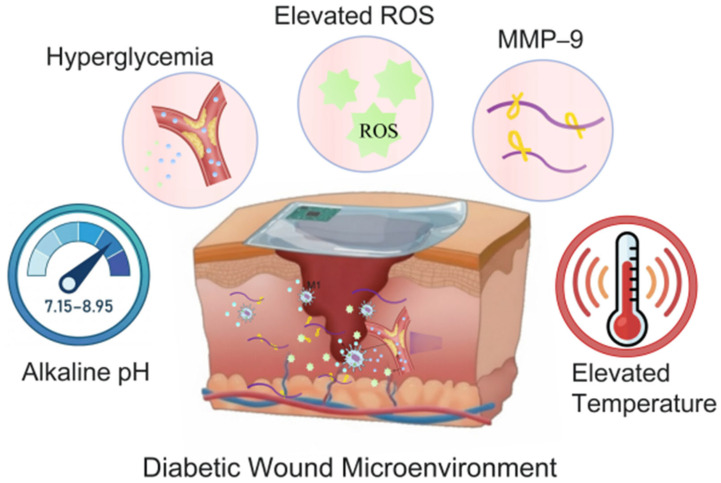
Schematic illustration of the diabetic wound microenvironment. Pathological factors, including hyperglycemia, elevated ROS, and overexpressed MMP-9, alongside elevated temperature and alkaline pH, collectively impede the healing process. (The arrows represent possible pathways. Gemini 3.0 pro was used to generate certain elements of this figure).

In addition to the biochemical and metabolic derangements discussed above, the physicochemical microenvironment of diabetic wounds also undergoes significant alterations. Regarding pH, in contrast to the slightly acidic environment of healthy skin (ranging from pH 4 to 6), chronic wounds typically exhibit a more alkaline milieu, with pH values ranging from 7.15 to 8.95, reflecting a broader spectrum of pH variation [40,41]. Temperature serves as another critical physical parameter. In normal wound healing, appropriate local temperature supports essential metabolic and enzymatic activities. In diabetic wounds, however, temperature alterations are commonly observed. Notably, a rise in local wound temperature is not directly caused by diabetes per se; rather, it primarily reflects the prolonged and dysregulated inflammatory response characteristic of diabetic wounds. This sustained inflammation, driven by persistent recruitment of neutrophils and macrophages and elevated levels of pro-inflammatory cytokines such as IL-1β and TNF-α, can contribute to a measurable increase in periwound temperature compared to acutely healing wounds. For instance, Armstrong et al. demonstrated that elevated local skin temperature often serves as an indicator of underlying tissue inflammation and may precede the clinical detection of early-stage ulcer development [42]. Thus, while temperature elevation alone cannot be interpreted as a direct marker of diabetes, it provides useful physiological insight into the chronic inflammatory state that impairs healing. Collectively, these intricate microenvironmental features establish the pathological foundation that obstructs diabetic wound healing. In summary, these complex biochemical derangements (hyperglycemia, ROS accumulation, and MMP-9 overexpression) and physicochemical anomalies (alkaline pH and temperature fluctuations) collectively constitute the pathological foundation that hinders diabetic wound healing (Figure 2). While traditional passive physical dressings continue to meet clinical needs in a substantial proportion of diabetic wound cases, the growing global burden of diabetic wounds—coupled with the rapid advancement of sensing and monitoring technologies—offers considerable opportunities for further improvement beyond current standards of care. In this context, the development of intelligent diagnostic and therapeutic platforms capable of real-time sensing of the aforementioned key biomarkers and providing on-demand intervention represents a promising direction for future research. Such intelligent platforms must first possess the interfacial stability required to withstand this hostile microenvironment to subsequently achieve high-fidelity, in situ dynamic monitoring of these critical biomarkers.

## 3. Stability and Microenvironment Regulation of Wound-Sensing Interfaces

The clinical translation of continuous wound monitoring technology has long been hindered by the complex biochemical and physical microenvironments characteristic of diabetic wounds. In practical operation, sensors face multifaceted challenges, including detachment due to mechanical strain, biofouling from exudates, short-circuiting caused by hypertonic fluids, and signal interference arising from oxidative stress. To ensure high-fidelity and long-term stability of monitoring signals, the design of sensing interfaces has evolved beyond simple passive protection toward multi-layered active microenvironment remodeling across physical, fluidic, and biochemical dimensions.

### 3.1. Tissue Adhesion and Mechanical Stability of Flexible Interfaces

Because diabetic wound sites are frequently subjected to high-frequency cyclic pressure and frictional shear forces, the physical attachment of conventional flexible sensing interfaces is highly susceptible to delamination or fatigue failure. This remains the primary cause of interruptions in continuous monitoring.

To enhance interfacial adhesion stability, researchers have utilized active groups (such as polyphenol structures) within biomimetic or natural polymer chains to establish robust chemical or physical anchoring at the interface between the hydrogel dressing and the tissue, replacing traditional passive physical attachment. For example, by incorporating tannic acid (TA) into zwitterionic hydrogels, the adhesive properties of the polyphenol groups can significantly increase the hydrogel’s skin adhesion to 20.2 kPa [43]. This interfacial reinforcement mechanism effectively offsets shear stresses generated by plantar (sole of the foot) activities, ensuring tight conformal contact between the hydrogel and the wound bed. Furthermore, composite networks constructed using polydopamine (PDA) functionalized nanoparticles have also achieved a tissue adhesion strength of 14.7 kPa [44].

To address the issues of signal interruption or structural failure caused by high-frequency heavy loading, researchers have enhanced the damage resistance and self-healing capabilities of materials by constructing dynamic nanocomposite networks. For instance, Fang et al. incorporated tannic acid (TA) into zwitterionic poly (sulfobetaine methacrylate) (polySBMA) hydrogels to develop a mechanically reinforced hydrogel with diverse biological functions. Experimental results demonstrate that, taking TA-reinforced zwitterionic hydrogels as an example, such reinforced interfaces can withstand over 3500 cycles of cyclic compression at approximately 200 kPa (equivalent to the maximum in-shoe plantar pressure of the human body) without structural failure, indicating robust durability under repetitive mechanical loading conditions simulating approximately 3500 gait cycles (steps) during walking. This ensures the continuity of monitoring in high-load applications [43]. Additionally, the introduction of vinylimidazole monomers can further enhance network strength through metal ion chelation [45].

### 3.2. Directional Drainage of Wound Exudate and Anti-Maceration Design

In addition to mechanical challenges, the accumulation of high-volume exudate and tissue overhydration represent the primary physical obstacles facing flexible sensors and dressings. If continuously secreted high-viscosity exudate is allowed to stagnate at the contact interface, it not only causes maceration damage to the surrounding healthy tissue but may also form a highly conductive liquid film that triggers short-circuiting of the sensing channels. Traditional homogeneous hydrogels often struggle to balance efficient absorption with mechanical stability, as they are prone to excessive swelling.

**Figure 3:**
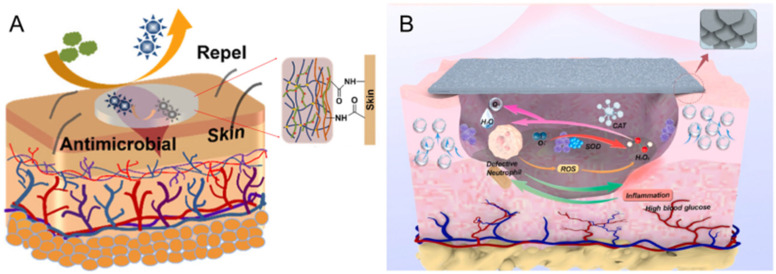
Interfacial regulation of the wound microenvironment. (**A**) Spatial decoupling design of a Janus hydrogel for anti-fouling and tissue adhesion [46]. (**B**) Mechanism of a superhydrophobic biocatalytic coating for in situ ROS scavenging [47].(The arrows represent enzymatic reactions).

To achieve functional decoupling, researchers have introduced asymmetric Janus structures, which synergistically resolve the conflict between interfacial adhesion and anti-fouling at the physical dimension. For example, Liu et al. [46] enhanced tissue anchoring by coating the contact side with chitosan while introducing a zwitterionic polymer network on the outward-facing side to block external bacterial attachment (Figure 3A). This spatial isolation design ensures that the dressing maintains stable adhesion in complex environments without compromising the biological cleanliness of the interface.

A more central breakthrough lies in the active unidirectional fluid management enabled by Janus structures. By constructing asymmetric wettability gradients (e.g., hydrophilic/hydrophobic differentials), materials can generate a Laplace pressure gradient that drives the unidirectional discharge of exudate. For instance, researchers developed an asymmetric structure by coating one side of a polyurethane sponge with superhydrophobic nanoparticles (such as F-ZnO@AgNPs). Combined with a near-infrared (NIR) photothermal effect, this achieved active, controllable unidirectional removal of exudate, creating an optimal moisture balance for the wound [48]. Based on the stable environment created by this directional transport mechanism, multifunctional Janus membranes can integrate sensing elements such as phenol red or Eu-MOF to achieve high-fidelity monitoring of wound pH (5–8) and $H_{2}O_{2}$ concentrations [49]. Recent studies have further extended fluid management to bidirectional intelligent exchange: through a three-layer electrospinning design, these systems can not only pump exudate away from the wound bed but also perform the controlled release of bioactive substances, such as silver ions or silicates. This dual functionality inhibits infection while simultaneously promoting angiogenesis (vascular regeneration) [50].

### 3.3. Anti-Biofouling and ROS Scavenging Mechanisms of Sensing Interfaces

#### 3.3.1. Zwitterionic Anti-Biofouling Interfaces

At the biochemical and signal-transmission levels, biofouling is a critical factor undermining the stability of wound sensors and dressings. The high-load protein exudate and bacteria characteristic of diabetic wounds are prone to non-specific adsorption on material surfaces, forming an insulating biofilm that leads to electrical signal attenuation or distortion. To address this, researchers have utilized zwitterionic polymers to construct contact interfaces with active anti-fouling properties.

For example, a 2022 study incorporated DMAPS (a zwitterionic monomer) into a cross-linked network, leveraging its anti-fouling characteristics to repel bacterial debris and provide a clean microenvironment for the wound bed [45]. Furthermore, the interfacial purification enabled by zwitterionic networks is a prerequisite for achieving high-fidelity multimodal monitoring. By eliminating interference from proteins and bacterial debris, the functional independence of individual sensing elements is preserved. A 2021 study utilized zwitterionic hydrogels to develop a sandwich-structured sensor system [51]; thanks to this anti-interference microenvironment, the system achieved continuous monitoring of temperature, strain, and glucose concentration while effectively avoiding crosstalk between multimodal signals [51].

#### 3.3.2. Nanozyme-Integrated ROS Scavenging Systems

In addition to physical and biofouling challenges, excessive reactive oxygen species (ROS) within the diabetic wound microenvironment are a key factor impacting interfacial stability. High concentrations of superoxide radicals and H_2_O_2_ not only trigger severe oxidative stress and delay healing but also generate excessive biochemical noise that interferes with potential future flexible electrochemical probes. To optimize the biochemical environment of the interface, researchers have constructed active chemical intervention systems by integrating high-efficiency nanozymes. The primary strategies include:

Interfacial Catalytic Purification: This strategy involves utilizing techniques such as magnetron sputtering to deposit metallic nanostructures with biomimetic enzyme activities onto the substrate. For instance, coatings based on vanadium-ruthenium-boron (VRuB) intermetallic compounds [47] exhibit exceptional catalase-like (CAT-like) activity, with a maximum reaction rate (V_max_) reaching $48.53\times 10^{-6}$ Ms^−1^. This design efficiently scavenges ROS (Reactive Oxygen Species) while simultaneously maintaining superhydrophobic anti-adhesion properties at the contact interface (Figure 3B).

3D Network Remodeling: Beyond surface coatings, researchers are integrating nanozymes directly into 3D hydrogel networks to achieve deep-tissue microenvironment modulation. For instance, the incorporation of polydopamine-coated hollow manganese dioxide nanoparticles(hMnO_2_@PDA NPs) [52] endows the hydrogel with dual superoxide dismutase (SOD)-like and catalase (CAT)-like activities. This multi-enzyme mimetic capability not only scavenges ROS but also converts them into O_2_, thereby alleviating wound hypoxia. Furthermore, it mitigates inflammatory responses by modulating macrophage polarization (typically promoting the transition from the pro-inflammatory M1 phenotype to the pro-healing M2 phenotype).

In summary, by integrating flexible anti-biofouling designs, Janus-structured fluid management, and targeted biochemical purification of ROS, researchers have successfully constructed multifunctional dressings and sensing interfaces with highly stable physicochemical properties. This robust interface serves as both a physical and chemical shield, protecting sensors against the harsh conditions of the wound environment. More importantly, by mitigating background noise and fluidic interference, it establishes a critical foundation for acquiring high-fidelity signals. In essence, such interface stability is not merely a protective measure but a prerequisite; without it, the accuracy and reliability of any downstream multimodal sensing would be fundamentally compromised. With this “hardware” security in place, the focus of intelligent diagnosis and treatment now shifts toward achieving high-sensitivity, multimodal in situ monitoring of core diabetic wound biomarkers.

## 4. In Situ Monitoring of Key Wound Parameters

Having addressed fundamental challenges such as interface failure and signal drift, the research focus of smart dressings has shifted toward leveraging diverse sensing mechanisms to achieve a precise deconstruction of pathological processes. Built upon stable flexible interfaces, current monitoring systems have evolved into sophisticated networks encompassing optical signaling, electrochemical quantification, and multimodal integration. These systems aim to reveal the underlying biological mechanisms of healing arrest in chronic diabetic wounds through the high-fidelity tracking of core biochemical indicators, such as glucose metabolic balance, oxidative stress levels, and protease activity.

To provide a systematic overview, this section follows the hierarchical nature of wound pathophysiology: we first discuss physicochemical parameters (pH and temperature) as broad indicators of infection, then metabolic parameters (glucose) reflecting underlying dysregulation, and finally inflammation-specific biomarkers (ROS and MMP-9) as direct molecular drivers of stalled healing. This progression from general cues to specific mediators mirrors clinical diagnostic logic.

Table 1 systematically summarizes the key performance metrics of recent sensor-integrated dressings, detailing their detection limits, sensitivities, response times, and stabilities across relevant healing stages.

### 4.1. Monitoring of Wound pH Levels

Given that the alkaline shift within the wound microenvironment is typically the earliest chemical hallmark of infection risk, establishing rapid and intuitive screening methods is crucial for early intervention [53,54]. Currently, various monitoring strategies have been developed, ranging from qualitative colorimetric screening to integrated detection technologies based on fluorescence analysis.

**Figure 4:**
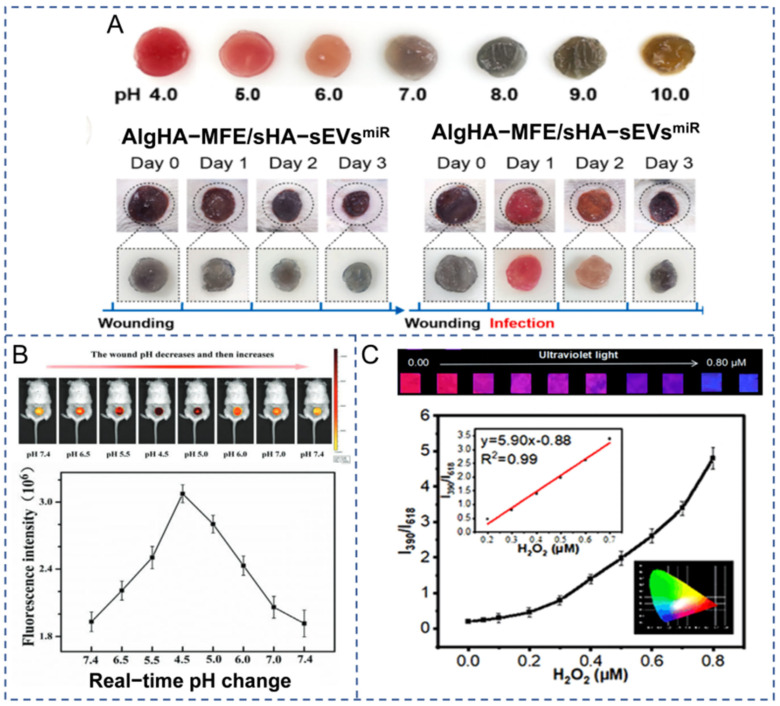
Multimodal optical monitoring strategy for wound microenvironment. (**A**) Hydrogel dressing based on natural colorimetric dye (mulberry extract, MFE) [55]. (**B**) The application of hydrogels equipped with near-infrared (NIR) fluorescent probes in in vivo wound models demonstrated the ability to monitor the real-time changes in fluorescence signal intensity reversibly with pH (from 7.4 to 4.5 and then back to 7.4) [60]. (**C**) Ratio fluorescence (self-calibrated) monitoring technology for H_2_O_2_ detection. The dressing exhibits a fluorescence transition from red to blue at different H_2_O_2_ concentrations, and its fluorescence intensity ratio I390/I618 shows a good linear relationship with H_2_O_2_ concentration (0.05–0.80 µM), which improves the accuracy of detection [49].

Among existing strategies, colorimetric sensors have become the preferred method for visual triage due to their ability to directly convert invisible chemical changes into color transitions perceptible to the naked eye. Typically, these sensors function by integrating pH-responsive indicators into the dressing matrix. To enhance biocompatibility and safety, researchers have extensively investigated pH indicators derived from natural pigments. For instance, anthocyanins—natural pigments extracted from plants such as mulberry [55], red cabbage [56], and cyanidin [57]—have been successfully incorporated into substrates like chitosan and alginate. As illustrated in Figure 4A, these dressings exhibit distinct colorimetric responses across various pH levels [55]. Under acidic conditions (pH 4.0–6.0), they transition through a spectrum of red, pink, and orange. In contrast, under weakly alkaline conditions (pH 8.0), the color shifts to blue-gray, while further increases in alkalinity trigger subsequent transitions to dark green or yellowish-brown. This pronounced color gradient serves as an immediate visual alarm, acting as an initial non-invasive screening step to minimize unnecessary dressing removals. Beyond natural dyes, synthetic dyes such as phenol red [53] and α-naphtholphthalein [54] have also been utilized in dressing development for their superior pH sensitivity. For example, Godau et al. [54] developed a calcium alginate hydrogel dressing incorporating α-naphtholphthalein, which responds to pH fluctuations in wound exudate with clear color shifts. Rather than providing a definitive diagnosis of infection, this colorimetric response is used to classify the risk of infection into low, moderate, and high levels based on specific pH thresholds (e.g., pH 7.75 for moderate risk and pH 8.3 for high risk). The clinical significance of this system lies in remote triage: it allows low-risk wounds to be monitored remotely, thereby safely avoiding unnecessary dressing removals. However, for wounds classified as moderate to high risk, closer monitoring and clinical intervention in the form of dressing removal and wound culture are still strictly required to identify the specific pathogen.

Despite their strengths in qualitative screening, colorimetric sensors often lack the resolution required for precise quantification and can be limited by the subjectivity of the naked eye. To enhance diagnostic accuracy and capture subtle physiological fluctuations, fluorescence analysis has garnered significant attention. Current methodologies utilize a diverse array of fluorescent probes—including rare earth complexes [58], carbon dots (CDs) [59], and organic fluorescent dyes [60]—to transduce pH variations into detectable optical signals.

For rigorous quantitative analysis, Zhang et al. [59] achieved a linear fluorescence response across a pH range of 5.0–9.5 using metal–organic framework nanofibers encapsulated with carbon dots (GOx/CDs@MOFNFs). In this system, the CDs function as pH-sensitive indicators; the abundance of acidic and basic functional groups on their surface enables a highly sensitive response to hydrogen ion concentration shifts within the microenvironment. Under acidic conditions, high hydrogen ion concentrations induce the protonation of these surface groups, yielding robust blue fluorescence (emission peak at 435 nm) under 365 nm UV excitation.

Conversely, as the environmental pH rises, the decreasing hydrogen ion concentration leads to the deprotonation of the functional groups. This process triggers fluorescence quenching, resulting in a marked decrease in blue fluorescence intensity. Experimental data demonstrated an excellent linear correlation between the blue channel values and pH levels (R^2^ = 0.9909), allowing for the precise conversion of colorimetric data into digital pH values. This facilitates the in situ, real-time assessment of the wound microenvironment in diabetic wounds. Even under complex lighting conditions, this fluorescent probe approach maintains exceptional sensitivity and demonstrates superior quantitative capabilities.

To extend these applications to deep-tissue monitoring within the human body, Zong et al. [60] utilized near-infrared (NIR) fluorescent probes (Figure 4B). The longer emission wavelengths of NIR probes not only facilitate deeper tissue penetration but also significantly minimize biological autofluorescence, ensuring high-precision in vivo pH imaging that exceeds the capabilities of surface visual inspection.

In summary, the evolution of wound pH monitoring technology reflects a strategic transition from intuitive, qualitative visual triage toward high-fidelity, digital quantitative analysis. Natural and synthetic colorimetric sensors provide a convenient and biocompatible first-line defense for infection screening, while the emergence of fluorescent probe technology addresses the core requirement for objective and precise measurement of the wound microenvironment. This multi-layered monitoring framework not only captures real-time biochemical fluctuations during the healing process but also establishes a robust data foundation for the advanced digital diagnostics and personalized therapeutic interventions discussed in subsequent chapters.

### 4.2. Monitoring of Wound Temperature

Given that local hyperthermia is a critical physical indicator of inflammation or infection, establishing an effective thermal warning system is equally essential for diabetic wound management, serving as a complementary tool to pH monitoring. To provide patients with immediate visual alerts, strategies based on structural color or thermochromic materials have garnered significant attention.

**Figure 5:**
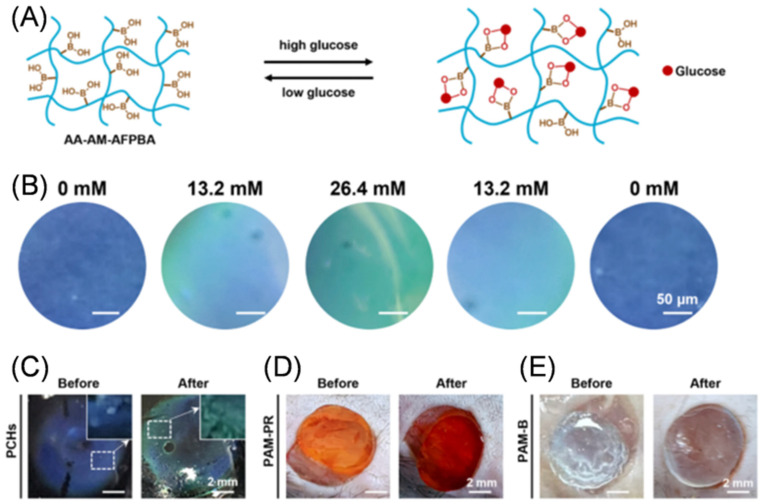
Representative examples of smart hydrogel dressings for visual monitoring of wound microenvironment indicators. (**A**) Schematic illustration of the glucose-responsive mechanism, where competitive binding of AFPBA with glucose triggers hydrogel swelling. (**B**) In vitro visual readout of photonic crystal hydrogels (PCHs) showing intuitive structural color changes across a physiological glucose range (0–26.4 mM), where different colors represent different glucose concentrations. (Scale bar = 50 μm). (**C**–**E**) In vivo monitoring of diabetic wound biomarkers in a mouse model, comparing the dressing appearance “Before” and “After” application (Scale bar = 2 mm): (**C**) hyperglycemia detection via PCH color shift, (**D**) alkaline environment alert using pH-responsive indicator, and (**E**) infection-related hyperthermia warning (>38 °C) via thermochromic powders. Images adapted from Ref. [61] under the terms of the Creative Commons CC-BY-NC-ND license.

A notable example is the study by Yang et al. [61], who successfully developed a visual “fever alert” for a critical wound temperature of 38 °C by integrating specific thermochromic powders into polyacrylamide hydrogels. Under normal body temperature conditions, the dressing appears blue; however, when the local wound temperature exceeds 38 °C due to bacterial infection or inflammation, the dressing rapidly turns colorless within 15 s (Figure 5A). Even after multiple heating and cooling cycles, these thermosensitive hydrogel modules maintain excellent reversibility and stability. Rather than serving as a definitive diagnostic tool, this intuitive response mechanism acts as a critical early warning for an amplified inflammatory response or potential infection, offering diabetic patients a simple method to recognize abnormal thermal status and seek timely clinical assessment.

While visual alerts provide a highly accessible qualitative indication, localized hyperthermia can occur during both the natural inflammatory phase of wound healing and acute infection. Consequently, clinical management often requires continuous, high-resolution monitoring to distinguish between routine sterile healing and prolonged infectious states.

To supplement qualitative visual indicators with high-precision quantitative data, resistive temperature sensors have been extensively developed. These sensors typically utilize thermoresponsive materials to transduce subtle temperature fluctuations into measurable electrical signals. For instance, Guo et al. [51] developed a hydrogel system based on the thermosensitive phase transition of N-isopropylacrylamide (NIPAAm), achieving a high-sensitivity resistive response within the physiological temperature range of the wound (35–40 °C). As the temperature rises, the NIPAAm segments within the hydrogel network undergo a thermally induced phase transition, thereby enhancing the hydrophobic interactions between NIPAAm molecules. This transition leads to an increase in ion concentration within the hydrogel, which subsequently causes a decrease in the material’s electrical resistance. Such devices facilitate the continuous and precise monitoring of temperature variations, providing the critical temporal data needed to identify persistent thermal plateaus or secondary spikes—key signatures that differentiate severe infection from natural inflammation in complex diabetic wounds.

### 4.3. Monitoring of Wound Glucose Levels

Beyond assessing the physicochemical parameters of the wound (such as pH and temperature), wound status can also be evaluated at the metabolic level through key biomarkers, most notably glucose. While systemic blood glucose monitoring (e.g., finger-prick tests or continuous glucose monitors) is universally accessible and remains the gold standard for overall diabetic management, it may not perfectly reflect the localized metabolic reality of a chronic wound. In diabetic ulcers, severe microvascular impairment, localized ischemia, and bacterial consumption can create significant discrepancies between systemic plasma glucose and the actual glucose concentration within the local wound bed. As previously discussed, localized hyperglycemia is not only a primary obstacle to wound healing in diabetic patients but also a precipitating factor for oxidative stress, inflammatory responses, and impaired angiogenesis. Consequently, integrating glucose-sensing capabilities into smart dressings facilitates the real-time monitoring of glucose fluctuations, providing critical early warnings for potential metabolic dysregulation and assisting clinicians in timely, precise interventions. To meet these diverse clinical requirements, current research has progressed beyond simple detection, establishing a comprehensive monitoring ecosystem that spans the transition from traditional colorimetric arrays to high-precision, non-invasive, and multimodal sensing frameworks.

Optical visualization strategies offer unparalleled convenience for patient self-monitoring and rapid screening. Mirani et al. [62] developed a multifunctional sensing platform that integrates enzymatic sensors, utilizing a glucose-induced colorimetric reaction (transitioning from pale yellow to deep red) to indicate concentration levels. This provides qualitative feedback on wound glucose levels through intuitive color transitions. Furthermore, Yang et al. [61] leveraged the physical properties of photonic crystal (PC) structures to generate structural color changes driven by glucose-induced swelling or contraction. As depicted in Figure 5B, the double-network PCHs exhibit distinct structural color shifts across the physiological glucose range (0–26.4 mM), enabling a direct visual readout of metabolic status without external power. Experimental results demonstrated that the double-network (DN) PC hydrogel achieves a response in just 15 min, doubling the response speed compared to single-network structures. These optical strategies not only define the sensitivity boundaries of monitoring but also provide standardized visual signal flows for subsequent digital feature extraction.

However, to transition from discrete patient examinations to continuous remote disease management, wireless electrochemical sensing has become indispensable. To overcome the limitations of optical readouts and facilitate remote monitoring, electrochemical sensing is widely adopted for its exceptional sensitivity and direct signal transduction—typically via current [63] or potential [64]. Gu et al. [64] developed a self-powered patch utilizing laser-scanning technology for the in situ fabrication of laser-induced nanozyme electrodes, thereby constructing a highly stable nanozyme-based glucose biofuel cell (BFC). This BFC utilizes the open-circuit voltage (EOCV) to monitor wound glucose levels in real time. The EOCV of the device demonstrates a strong linear relationship (R^2^ = 0.996) with glucose concentrations in the range of 0 to 20 mM, with a sensitivity of 10.9 mV mM^−1^. Such a configuration not only achieves high-specificity detection but also enables the integration of wound care into the Internet of Things (IoT), establishing a solid foundation for telemedicine and closed-loop biomedical systems.

While the aforementioned strategies excel in surface-level monitoring, they typically lack the capacity to spatially map the metabolic landscape within deep tissues—a capability essential for comprehensive wound management. To bridge this gap and achieve non-invasive deep-tissue diagnostics, Han et al. [65] proposed a unique monitoring scheme tailored for clinical settings. They utilized magnetic resonance imaging (MRI), a highly advanced medical imaging modality, to develop a multifunctional diagnostic hydrogel system. Within this system, the dual functionality of the GOx–MnO_2_ nanozymes arises from their catalytic synergy, which operates through an efficient two-step cascade reaction. First, GOx acts as the recognition element, selectively oxidizing glucose to produce gluconic acid and H_2_O_2_. However, H_2_O_2_ is a toxic reactive oxygen species (ROS) that can exacerbate inflammation in diabetic wounds. At this stage, the MnO_2_ nanozyme plays a synergistic role by mimicking catalase, rapidly decomposing the locally generated H_2_O_2_ into water and O_2_. This cascade enables genuine dual functionality: on one hand, it removes ROS while generating O_2_, thereby alleviating oxidative stress and local hypoxia to promote therapeutic outcomes; on the other hand, MnO_2_ is reduced during the reaction to release paramagnetic Mn^2+^ ions. These Mn^2+^ ions serve as effective contrast agents for T1-weighted MRI, significantly enhancing imaging signals directly correlated with glucose depletion. Consequently, this cascade elegantly converts metabolic activity into high-resolution, tomographic visualization of glucose metabolism, enabling real-time, quantitative monitoring of the wound microenvironment.

### 4.4. Monitoring of Wound ROS

Complementing general physicochemical parameters, the accurate assessment of inflammatory severity necessitates the monitoring of specific pathological mediators. Chronic hyperglycemia frequently precipitates oxidative stress, leading to the overproduction of ROS, including hydrogen peroxide (H_2_O_2_) and hypochlorous acid (HClO). These reactive molecules serve as vital biomarkers within the inflammatory process; their in situ concentration fluctuations provide a biochemical signaling foundation for the clinical evaluation of inflammation levels and the dynamic adjustment of therapeutic regimens.

To enable rapid qualitative assessment of oxidative stress, Huang et al. [66] developed a multifunctional dressing incorporating oxygen-deficient molybdenum trioxide (MoO_3−x_) nanosheets. Within this system, MoO_3−x_ functions as a highly sensitive colorimetric probe that undergoes a redox reaction in the presence of H_2_O_2_, triggering a color transition from blue to colorless. However, given the complex optical properties of wound exudate, relying solely on absolute intensity fluctuations can be prone to interference. To achieve reliable quantitative analysis through signal self-calibration, Fluorescence Resonance Energy Transfer (FRET) technology offers a robust alternative. Cui et al. [67] designed a FRET-based dressing utilizing graphene quantum dots (GQDs) as donors and luminescent porous silicon (PSi) as acceptors. In this configuration, H_2_O_2_ oxidatively degrades the PSi acceptor, thereby disrupting the FRET effect. This disruption results in the attenuation of red fluorescence and the recovery of blue fluorescence. This dual-color ratiometric mechanism, comprising red and blue emissions, establishes an inherent self-calibration signal at the biochemical level. By effectively counteracting background noise at the sensing source, it ensures the robustness of optical signals within the complex wound fluid environment. Employing a similar ratiometric strategy, Liu et al. [49] utilized a dual-emission fluorescence system to achieve a specific optical response to H_2_O_2_ by tracking the intensity ratio at 390 nm and 618 nm, providing a precise signal foundation for subsequent quantitative conversion (Figure 4C).

Although H_2_O_2_ serves as a broad indicator of oxidative stress, targeting specific ROS components allows for a more profound understanding of active immune defense mechanisms. Among these, HClO possesses particular diagnostic value as a pivotal inflammatory biomarker across multiple pathological stages. Zong et al. [68] engineered a functionalized ionic liquid-hyaluronic acid hydrogel, incorporating a sulfide-modified near-infrared (NIR) fluorophore, SCy-7, via covalent grafting to achieve sensing functionality. In this system, SCy-7 acts as the specific recognition moiety; upon exposure to elevated concentrations of HClO within the wound microenvironment, its conjugated structure is disrupted through potent oxidation. This process results in significant fluorescence quenching at 778 nm (a “signal-off” response), enabling real-time imaging and the specific assessment of inflammation levels in diabetic wounds.

**Table 1:** Summary of recent sensor-integrated dressings for monitoring diabetic wound biomarkers.

Biomarker(s)	Relevant Healing Stage	Sensing Materials and Recognition Element	Range/LOD	Sensitivity/Linearity	Response Time	Stability/Lifetime	Ref.
Temp, Strain, Glucose	Inflammation and proliferation phases	SBMA hydrogel, NIPAAm, MPBA	Temp: 25–65 °C Strain: 3–11.95% (Min LOD: 0.25 kPa) Glu: >20 mmol/L	Temp: Quadratic function (R^2^ > 0.99)	-	12 days (in vivo)	[51]
pH	All stages of diabetic wound healing	GelMA/CMCSMA hydrogel, Phenol red, GACo MPNs	pH 5.0–9.0	ML prediction accuracy: 96%	Rapid	21 days (in vivo)	[53]
pH	Healing and infection stages	Alginate hydrogel, α-naphtholphthalein	pH 7.5–8.5	ML classification accuracy: 98.1%	~20 min	14 days (in vivo)	[54]
pH	Inflammation and infection stages	Alginate/HA hydrogel, Anthocyanins (MFE)	pH 4.0–10.0	Color gradient (red to blue-gray)	6–24 h (in vitro)	14-day sustained release	[55]
pH	All stages of diabetic wound healing	Hydrogel matrix, Anthocyanins	pH 5.0–9.0	-	Minutes	8–13 days (in vivo)	[57]
pH	All stages of diabetic wound healing	Eu-EDTA complexes	pH 4.5–7.5	RGB red channel (R^2^ = 0.9995)	1 min	72 h (in vitro)	[58]
pH	All stages of diabetic wound healing	Cu-MOF nanofibers, Carbon dots (CDs)	pH 5.0–9.5	-	Rapid	>10 reversible cycles	[59]
pH	All stages of diabetic wound healing	PIL/CS hydrogel, CyO NIR probe	pH 4.5–7.4	-	Real-time	Stable for up to 30 days	[60]
Glucose, pH, Temp	All stages of diabetic wound healing	Photonic Crystal (PC), Phenol red (PR) or Bromophenol blue (BB)	Glu: 0–26.4 mM pH: 7.0–9.0 Temp: ~38 °C	Significant Hue shifts	pH: 5–15 s Temp: ~15 s Glu: 15 min	5–8 reversible cycles	[61]
pH, Glucose	All stages of diabetic wound healing	Alginate hydrogel, Phenol red, GOx/HRP	pH: 4.0–9.0 Glu: 0–12 mM	pH: Accuracy ±4% (alkaline), ±6% (acidic) Glu: Red + green channel (R^2^ = 0.98)	pH: <35 min Glu: Real-time monitoring	1 month storage at −20 °C	[62]
pH, Glucose	All stages of diabetic wound healing	PANI electrode, Au/Fc-PEI/GOx	pH: 3.0–9.0 Glu: 0–22 mM (LOD: 0.097 mM)	pH: 62 mV/pH Glu: 0.48 µA/(mM·cm^2^)	30 s	Stable operation for >24 h	[63]
Glucose, pH	All stages of diabetic wound healing	Au/CuS-LIG, Pt-LIG, PANI-LIG	Glu: 0–20 mM pH: 4.0–9.0	Glu: 10.9 mV/mM pH: 52.9 mV/pH	75 s (pH)	>30 days shelf life	[64]
Glucose	Inflammation, proliferation, and remodeling phases	HA hydrogel, GOx-MnO_2_ nanozymes	Glu: 3.1–100 mM	Highly linear (R^2^ = 0.9923, MRI)	~1 h (in vivo)	>14 days (in vivo)	[65]
pH, H_2_O_2_	All stages of diabetic wound healing	SA hydrogel, EuBG, MoO_3_ nanosheets	pH: 4.0–8.0 H_2_O_2_: 0–200 µM	pH: R^2^ = 0.99 H_2_O_2_: R^2^ = 0.992	10 min (H_2_O_2_)	14 days (in vivo)	[66]
H_2_O_2_ pH	All stages of diabetic wound healing	Chitosan film, GQDs/PSi nanochannels	H_2_O_2_: 0.1–10 mMpH: 6.5–7.4	-	1–24 h	1–4 days per dressing	[67]
HClO	Inflammation phase	HA/PIL hydrogel, SCy-7 NIR probe	LOD: 1 µM	-	5 min (in vivo)	3 days per dressing	[68]
MMP-9	Inflammation and proliferation phases	GelMA hydrogel, Peptide crosslinker	10–1000 ng/mL LOD: 8.9 ng/mL	Highly linear (R^2^ = 0.9866)	2 h	Stable for >4 days at 37 °C	[69]

Abbreviations: LOD, limit of detection; Temp, temperature; Glu, glucose; ML, machine learning; NIR, near-infrared; MRI, magnetic resonance imaging; GelMA, methacrylated gelatin; HA, hyaluronic acid; SA, sodium alginate; CS, chitosan; PAM, polyacrylamide; PANI, polyaniline; GOx, glucose oxidase; HRP, horseradish peroxidase; CDs, carbon dots; GQDs, graphene quantum dots; LIG, laser-induced graphene.

### 4.5. Monitoring of Wound MMP-9

Unlike the monitoring of upstream triggers such as glucose and ROS, the assessment of Matrix Metalloproteinase-9 (MMP-9) directly targets the downstream pathological effector molecules responsible for extracellular matrix (ECM) degradation and tissue necrosis [69,70,71]. Consequently, MMP-9 levels provide a more accurate real-time reflection of the wound’s actual remodeling status, offering critical data to guide timely therapeutic interventions.

To transduce this specific enzymatic activity into wirelessly readable signals, Deng et al. [69] developed an innovative bioelectronic sensor based on a flexible inductive-capacitive (LC) resonant circuit integrated with an MMP-9-responsive bioactive hydrogel. Unlike active electrochemical sensors, which typically require internal power sources or wired connections, this passive LC design operates via dielectric modulation. The sensing mechanism is triggered when the MMP-9 enzyme cleaves specific peptide crosslinkers within the hydrogel network. This proteolytic cleavage induces hydrogel degradation, which alters the local dielectric environment near the electrode surface and modulates the overall capacitance of the LC system. These capacitive changes are subsequently converted into quantifiable shifts in the circuit’s resonant frequency, enabling battery-free, non-invasive, and in situ quantification of MMP-9 activity. The sensor demonstrates high linearity (R^2^ = 0.9915) and a limit of detection (LOD) as low as 8.9 ng mL^−1^. This performance spans the clinically relevant range of MMP-9 concentrations, confirming its practical utility in identifying high-risk chronic wounds.

### 4.6. Integrated Multiparameter Monitoring Strategies

While the single-analyte strategies discussed above provide isolated insights, diabetic wound healing is a complex, multifaceted process involving the coordinated regulation of inflammation, bacterial infection, angiogenesis, and cellular metabolism. Consequently, relying on a single biomarker often results in diagnostic ambiguity due to a lack of specificity [66,72]. To construct a comprehensive physiological profile, it is essential to develop multifunctional integrated platforms capable of simultaneous cross-referencing of multiple microenvironmental parameters.

To address the need for concurrent environmental and metabolic assessment, Zhu et al. [72] proposed an optical integration approach utilizing zwitterionic hydrogels. This system integrates a phenol red indicator with a glucose oxidase/horseradish peroxidase (GOx/HRP) dual-enzyme system, enabling the simultaneous colorimetric detection of pH (4–8) and glucose (0.1–10 mM). To extend this integration from point sensing to spatial mapping, Mirani et al. [62] applied similar colorimetric principles to develop arrayed dressings. These arrays delineate the spatial distribution of pH and glucose within the wound, thereby enhancing the accuracy of assessing the heterogeneity of localized infections. Furthermore, to achieve a comprehensive “three-parameter” visual interface, Yang et al. [61] integrated photonic crystal structural color, thermochromic materials, and pH indicators onto a single dressing platform (Figure 5C). This integration enables the synchronized visual monitoring of glucose, temperature, and pH, generating intuitive color shifts at the material level that establish a crucial front-end hardware foundation for subsequent standardized RGB feature extraction and objective conversion via smart terminals.

**Figure 6:**
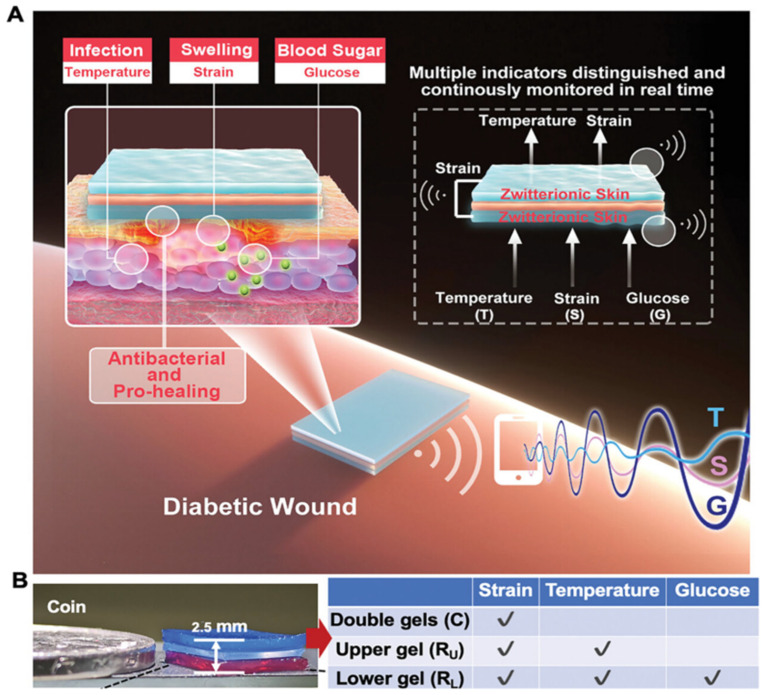
Multi-parameter integrated monitoring platform for avoiding signal crosstalk. (**A**) Schematic illustration of the sandwich-structured sensor based on multi-response zwitterionic skin for in situ multiple sensations (temperature, strain, and glucose) of diabetic wounds; the dashed rectangle highlights the sensing mechanism, and the white arrows represent the detection of various monitoring indicators. (**B**) Physical hierarchical design (double gels, upper gel, and lower gel) and its corresponding logic matrix for the stepwise decoupling of multimodal signals [51], where the red arrow indicates the corresponding relationship between the physical structure and the logic matrix.

In contrast to optical strategies that achieve integration by spatially separating indicators into distinct zones (arrays), creating compact electronic skins (e-skins) to meet clinical demands for automated, continuous monitoring often requires integrating multi-stimuli responsiveness into a single sensing unit. However, this high-density integration introduces a pivotal challenge well-documented in e-skin research: inevitable signal crosstalk. Unlike spatially isolated optical spots, a single sensing unit may simultaneously respond to temperature, biochemicals, and mechanical strain, resulting in convoluted outputs where signals are indistinguishable. To address this “multistimuli discrimination” challenge—arising from overlapping physical mechanisms within the sensor—Guo’s team [51] developed a sandwich-structured zwitterionic skin sensor designed specifically for stepwise signal decoupling (Figure 6). This configuration strategically differentiates the responsiveness of each layer: capacitance responds exclusively to mechanical strain (swelling); the upper resistance responds to both strain and temperature (infection); and the lower layer detects strain, temperature, and glucose. By leveraging the capacitive signal to filter strain artifacts and the upper resistance to calibrate for temperature, the system successfully isolates the glucose signal from the complex background. Specifically, the insulating elastomer layer isolates the top hydrogel from wound exudate, rendering it insensitive to glucose. As a result, the capacitive layer responds only to mechanical deformation, independent of temperature and glucose, enabling precise strain quantification. This quantified strain can then be subtracted from the resistance signal of the top layer to extract the pure temperature component. Subsequently, by removing both strain and temperature contributions from the bottom hydrogel, which is in direct contact with the wound and responds to all three stimuli, a glucose-specific signal can be obtained without cross-interference. This combined structural and circuit-level decoupling strategy effectively suppresses crosstalk among multiple parameters, enabling accurate quantitative sensing in complex environments.

Despite these advancements, the in situ acquisition of multidimensional pathological signals alone is insufficient to provide direct clinical guidance. Whether due to the susceptibility of optical sensors to visual subjectivity or the inherent complexity of multimodal electrophysiological signals, manual interpretation faces significant bottlenecks. Consequently, the digital transformation of raw physicochemical signals—facilitated by portable terminals and data analysis algorithms for physiological feature extraction—represents a pivotal step in advancing smart wound management toward practical clinical application.

Furthermore, to elucidate the selection criteria for these diverse integration strategies, a critical comparison of the underlying sensing technologies, detailing their respective advantages, limitations, and preferred clinical applications, is provided in Table 2.

## 5. Digitization and Intelligent Analysis of Monitoring Signals

Advanced wound sensing has made the in situ capture of multi-dimensional biomarkers a reality. Nevertheless, raw signals from flexible dressings often suffer from environmental noise and multimodal cross-interference, hindering direct clinical application. Deep digitalization and intelligent processing are essential to convert this complex underlying data into objective diagnostic metrics. This workflow begins with the standardized acquisition and quantification of signals using portable devices, followed by the application of machine learning for automated extraction and evaluation of high-dimensional wound features. This concludes in a scientific framework that supports precise clinical interventions.

### 5.1. Smartphone-Based Signal Acquisition and Quantification

The precise quantification of pathological signals from wounds is fundamental to achieving objective clinical evaluations. Historically, monitoring via dressings has been tethered to visual interpretations of colorimetric or fluorescent shifts. Such qualitative methods are notoriously prone to ambient light fluctuations and inter-observer variability, which compromises the consistency of clinical assessments [62]. To circumvent these visual biases, digitized acquisition of in situ optoelectronic signals via portable intelligent terminals has become the prevailing approach. In this architecture, the smart terminal acts as more than a recording device; it enables the standardized extraction of color space metrics (such as RGB coordinates) [59,72]. By converting subjective visual cues into structured digital matrices, these devices provide the high-fidelity data necessary to drive advanced downstream diagnostic algorithms.

In the quantification of optical signals, initial strategies are typically based on the spectral emission characteristics of the sensing materials, where intensities from specific color channels are extracted for numerical fitting. For instance, Huang et al. employed probes emitting long-wavelength fluorescence, directly extracting the red (*R*) channel signal to quantify pH values via a binomial regression equation [66]. Similarly, research on the PDA@BPs system utilized linear fitting of R-channel intensity for pH monitoring [73]. Conversely, Zhang et al. targeted blue-emitting carbon dot probes by extracting the blue (B) channel for calculations [59]. While this “single-channel extraction method” is more objective than visual assessment, it remains highly susceptible to fluctuations in ambient lighting.

**Figure 7:**
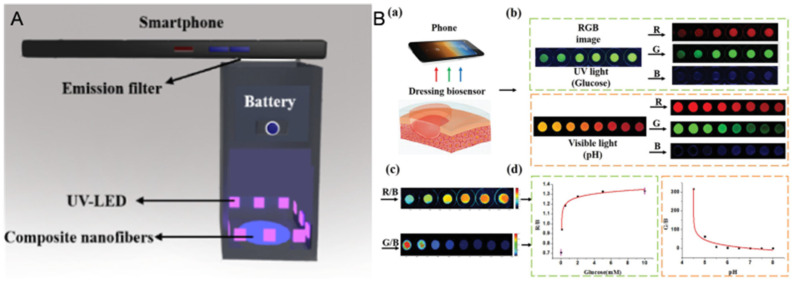
Strategies for standardized signal acquisition and anti-interference using smartphone terminals. (**A**) Schematic illustration of the hardware encapsulation strategy using a portable 3D-printed dark box equipped with a constant UV-LED light source and emission filter to eliminate ambient light fluctuations; the black arrows label the integrated device components. Adapted with permission from [59]. (**B**) The algorithm self-calibration (internal standard) strategy for multi-parameter monitoring and quantification. Adapted with permission from [72]. (**a**) The in situ signal acquisition process using a smartphone; the colored arrows represent the light pathways (red, green, and blue). (**b**) Extracted independent R, G, and B channel images under varying glucose concentrations and pH values. (**c**) Pseudo-color mapping images generated by calculating R/B and G/B ratios to automatically offset environmental light interference; the black arrows indicate the data processing flow. (**d**) Corresponding fitting curves (red lines) based on the experimental data points (black squares), enabling high-fidelity quantitative analysis of glucose and pH.

To mitigate environmental light interference, researchers have implemented hardware-based interventions. For example, Zhang et al. designed a 3D-printed portable darkbox equipped with an integrated UV-LED light source and optical filters, ensuring absolute constancy of the optical environment during image acquisition [59] (Figure 7A). To further enhance data precision, Al-Hawat et al. replaced smartphone cameras with a portable fluorometer, achieving an excellent linear response (R^2^ = 0.9909) within the clinical pH range of 6.0–9.0. Such rigorous hardware encapsulation not only minimizes interference but also enables the generation of high-definition spatial distribution maps of wound pH [74].

Currently, to move away from bulky physical darkboxes, the latest detection methodologies are shifting toward algorithmic self-calibration. Researchers have introduced an “internal standard” strategy: Zhu et al. utilized the relatively stable blue (B) channel as a reference, calculating R/B and G/B ratios to automatically offset errors caused by variations in light intensity (Figure 7B). By utilizing MATLAB-generated pseudo-color mapping and three-parameter logarithmic fitting equations, they achieved non-invasive monitoring of wound glucose and pH levels [72]. Similarly, Xie et al. leveraged smartphone recognition algorithms to establish anti-interference calibration curves for dual-detection based on R/B and B/R ratios [58].

In summary, the precision of optical feature extraction has been substantially improved through both hardware encapsulation and algorithmic self-calibration mechanisms. This high-fidelity, standardized front-end data quantification process not only ensures the reliability of monitoring signals within the complex wound environment but also lays a solid foundation for feeding these structured feature matrices into deep learning networks. Ultimately, this enables the realization of higher-dimensional automated diagnostics.

### 5.2. Wound Feature Extraction and Evaluation Based on Deep Learning

Although smartphone-based feature extraction (such as RGB channel analysis) enables the preliminary quantification of front-end signals, real-world clinical scenarios often present challenges that exceed the processing capabilities of simple linear fitting. These include the complex composition of wound exudates, non-uniform deformation of the dressing, and the potential coupling of multimodal signals. Consequently, the introduction of data-driven algorithms—epitomized by machine learning (ML)—has become a critical pathway for deciphering high-dimensional sensing data and eliminating residual subjective bias [75]. For instance, in the visual pH monitoring of smart hydrogel dressings, research has confirmed that the deep integration of these front-end visual parameters with smartphones and ML algorithms can significantly enhance the reliability of wound pH assessments and overall clinical management [53]. This shift signifies that wound management has evolved from simple “data collection” to a stage of “automated analysis” driven by machine vision. Machine learning bridges the gap between raw biophysical signals and clinical interpretation by capturing nonlinear relationships that linear models miss. Recent reviews have emphasized that AI is essential for data classification, noise reduction, and pattern recognition in multi-sensor systems [76]. By processing complex data such as RGB distributions, temporal variations, and sensor crosstalk, models like convolutional neural networks can extract subtle correlations and integrate them with existing pathological datasets for inference. In doing so, they convert noisy, fragmented signals into actionable clinical outputs, such as infection risk scores or healing stage classifications, enabling rapid triage decisions.

The integration of portable front-end hardware with cutting-edge back-end algorithms has established an efficient “hardware-algorithm” synergistic framework. For example, to overcome physical constraints—such as subjective color perception differences, ambient lighting fluctuations, and visual impairments associated with manual sensor reading—while addressing the time-intensive nature and psychological burden of continuous real-time monitoring, researchers have developed a machine learning (ML)-based smartphone application to interpret smart dressing colors and classify wound infection risks [54].

This application [54] automates infection detection via supervised learning, employing a four-step workflow: image acquisition, dressing detection, focused image analysis and classification, and result display. Utilizing the DenseNet201 Convolutional Neural Network (CNN) architecture, the classifier was trained on a balanced dataset comprising 7440 simulated images of both infected and non-infected states. The results demonstrated exceptional reliability and accuracy, with a mean classification accuracy of 98.1% ± 0.1%, while the True Positive Rate (TPR) and True Negative Rate (TNR) reached 98.9% and 97.4%, respectively.

In vivo validation studies indicate that these applications [54] do more than just facilitate diagnosis; they enable the objective stratification of patient infection risk, thereby allowing for proactive clinical triage. This data-driven automated framework represents a paradigm shift, transitioning traditional passive sensing dressings into comprehensive telehealth platforms. By doing so, it significantly reduces the reliance of wound management on specialized medical resources and effectively mitigates the burden on modern healthcare delivery systems.

## 6. Conclusions and Future Perspectives

This review summarizes how diabetic wound care has evolved from conventional passive dressings toward intelligent, real-time monitoring platforms. Owing to the hostile wound microenvironment characterized by hyperglycemia, excessive oxidative stress (ROS), and alkaline pH, diabetic wounds often exhibit severely impaired healing. Intelligent sensing systems enable accurate tracking of these key biomarkers, thereby providing clinicians with a clearer understanding of wound status. This article has discussed how the integration of stable sensor interfaces with intelligent data analysis can establish a complete closed-loop diagnostic system. Functionalities such as anti-biofouling design and active fluid management further ensure reliable sensor performance under harsh wound conditions. These advances make it possible to continuously and reliably monitor critical signals, including pH, glucose, ROS, and MMP-9. In addition, the use of smartphones and machine learning to convert visual colorimetric changes into quantitative digital outputs has greatly improved the objectivity and reliability of clinical wound assessment.

Nevertheless, a substantial gap remains between laboratory-scale validation of these devices and their practical implementation in clinical settings. To further advance this field, future research should focus on the following directions:

(1) Improving sensor stability and signal decoupling: Although current material strategies, such as zwitterionic polymers, help reduce biofouling, maintaining long-term sensing accuracy in complex wound exudates remains highly challenging. Future designs should develop more effective approaches for signal decoupling to minimize cross-interference during multiplexed detection, thereby ensuring accurate and reliable readouts.

(2) Moving from data acquisition toward AI-driven prediction and multimodal foundation models: At present, intelligent data analysis is mainly limited to signal calibration and classification of current infection risk. Future efforts should integrate continuous sensor outputs with deep learning algorithms to establish predictive models that identify early signs of delayed healing or infection, enabling proactive rather than passive interventions. Furthermore, the integration of large language models with multimodal AI opens new possibilities for wearable biosensors in wound care. In the future, such systems may combine continuous sensor data, wound images, and electronic health records to provide more complete assessments and support personalized treatment planning. However, moving toward clinical deployment remains challenging. On the technical side, improving the robustness and reliability of signal interpretation is still critical. On the ethical and practical side, issues such as data privacy in continuous monitoring, clinical responsibility, and the trustworthiness of AI-generated outputs need to be carefully addressed.

(3) Establishing standardized testing models: A large proportion of current studies still rely on simplified animal models, which cannot fully recapitulate the physiological characteristics of human diabetic skin. Standardized preclinical testing systems that better simulate human wound conditions and daily mechanical stresses are urgently needed. The establishment of such models would help accelerate the translation of intelligent wound dressings from laboratory research to real clinical application.

(4) Advancing toward fully autonomous intelligent systems: Beyond the current focus on sensing and data analysis, the next generation of intelligent wound platforms will likely integrate closed-loop therapeutic functions, where diagnostic information is automatically translated into on-demand treatment—such as triggered drug release, electrical stimulation, or photothermal therapy—without manual intervention. Achieving this vision will require seamless integration of interface intelligence, sensing intelligence, and diagnostic intelligence into a unified, autonomous system capable of real-time adaptation to the evolving wound microenvironment.

(5) Overcoming critical translational bottlenecks (Sterilization, Biocompatibility, and Costs): Beyond technical sensing performance, the practical transition of smart dressings from the bench to the bedside requires rigorously addressing specific clinical engineering challenges. Firstly, sterilization poses a significant hurdle. Smart dressings must withstand standard clinical terminal sterilization protocols (e.g., ethylene oxide gas, autoclave, or gamma irradiation) without degrading their physical and electronic properties. Due to their polymeric nature and the presence of high amounts of water, hydrogels and flexible conjugated polymers are generally highly sensitive to terminal sterilization. Processes like autoclaving or high-dose gamma irradiation can result in macroscopic degradation, polymer chain scission, or compromised bioactivity of incorporated sensing elements. Secondly, while initial cytocompatibility is frequently demonstrated in short-term laboratory settings, comprehensive long-term in vivo biocompatibility remains a paramount concern for clinical translation. The continuous exposure of the wound bed to integrated microelectronic components, heterogeneous nanomaterials, and hydrogel degradation byproducts necessitates rigorous evaluation to preclude secondary systemic toxicity or chronic foreign-body responses (FBR), which are often inadequately captured in preliminary studies. Finally, the cost of fabrication must be addressed. Current prototype development frequently relies on expensive laboratory techniques. Transitioning these complex smart dressings to scalable, cost-effective manufacturing processes is an absolute prerequisite to making these advanced healthcare platforms economically viable for widespread public health adoption. In summary, intelligent diagnostic platforms represent a new era in diabetic wound care. By transforming simple dressings into smart tools capable of supporting medical decision-making, these systems hold great potential to improve healing outcomes and reduce the risk of severe complications, including amputation. Achieving this goal will require continued interdisciplinary collaboration among experts in materials science, bioengineering, and medicine. As these intelligent systems become more reliable, practical, and user-friendly, they are expected to fundamentally reshape wound management, shifting the paradigm from passive observation to proactive and intelligent care.

## Figures and Tables

**Table 2:** Critical comparison of underlying sensing technologies for smart wound monitoring.

Sensing Technology	Modality	Working Principle	Key Advantages (Pros)	Limitations (Cons)	Preferred Clinical Application	Ref.
Optical	Colorimetric	Analyte triggers a visual color shift via an indicator.	Power-free, intuitive visual triage, high biocompatibility.	Lacks quantitative precision; susceptible to interference from the complex optical properties of wound exudate.	Rapid, first-line point-of-care screening for early infection.	[53,54,55,56,57,61,62,66]
Optical	Fluorescence	Analyte quenches or enhances emission signals.	High sensitivity; FRET technology enables signal self-calibration.	Probes are prone to photobleaching or chemical degradation.	Precise quantitative mapping of complex biochemicals (e.g., ROS).	[49,59,60,67,68]
Electronic	Electrochemical/Resistive	Direct signal transduction via current or potential changes.	Strong quantification capability, high linearity, and enables continuous tracking.	Faces multi-stimuli signal crosstalk, requiring complex structural and circuit-level decoupling strategies.	Continuous monitoring of specific metabolites (e.g., glucose) and temperature.	[51,63,64]
Electronic (LC)	Resonant	Biomarker alters the local dielectric environment or capacitance.	Battery-free, wireless detection, non-invasive signal transmission.	Measurement results are time-dependent (requires time-specific calibration curves).	Evaluating wound inflammation levels (MMP-9 concentration highly correlates with neutrophil counts).	[69]

Abbreviations: FRET, fluorescence resonance energy transfer; ROS, reactive oxygen species; LC, inductive-capacitive; RFID, radio frequency identification; NFC, near-field communication; MMP-9, matrix metalloproteinase-9.

## Data Availability

No new data were created or analyzed in this study.
